# How and Why Treatments Work in Psychiatry? Commentary: About the Irrationality of the Health Field

**DOI:** 10.3389/fpsyt.2018.00245

**Published:** 2018-06-07

**Authors:** Diogo Telles Correia, Massimiliano Aragona

**Affiliations:** ^1^Faculdade de Medicina, Universidade de Lisboa, Lisbon, Portugal; ^2^Crossing Dialogues Association, Rome, Italy

**Keywords:** validity, methodology, treatment, psychotherapy, theory of mind

In this article the author expresses a critical opinion regarding the state of research in the area of health care, particularly in the field of mental health.

It is stated that too much emphasis has been placed on efficacy studies, ignoring research on the theory behind treatments: “excessive focus on *what works* […], not also on *how and why it works* (e.g., underlying etiopathogenetic theory and/ or mechanism of change) introduced several major weaknesses and risks into the health field.” In particular, the risk is that by accepting treatments that are equally effective in clinical trials but based on untested or untestable theories, pseudo and non-scientific approaches or outdated treatments are introduced in the health system.

A solution would be to evaluate treatments not only for their efficacy but also on how much the theory on which they rely is supported (independently of the tests of treatment efficacy).

We agree that besides efficacy, it is relevant to know *how* and *why* a treatment works. Studies addressing *how* it works are part of those “good phenomenological analyses of what was once called the “psychotropism” of drugs (i.e., the drug induced modification of psychopathological phenomena)”[(1), p. 6]. Similar phenomenological analyses can be done for the effects of psychotherapies, basically relying on qualitative methods. However, David's article is silent on this, neglecting all inquiry on how treatments work. The focus is on the *why*, i.e., the point of the etiopathogenic chain modified by the treatment, or the mechanisms of change treatment-induced.

Here the author emphasizes the importance of the validity of the theory that bases treatments regardless of whether they are effective or not. Thus, to prove the validity of the theory, the major objective should, according to the author, be based on the etiopathogenesis of the disorder and the way the treatment acts in that etiopathogenesis.

Although the focus is on the mental health field, in David's article the most clear examples of etiopathogenesis come from general medicine (e.g., bacteria), where testing the point of the causal chain influenced by the treatment is easier.

However, validity in psychiatry can be of several types. To test the validity of a theory in psychiatry, the approach does not always have to be of the “realistic” type, claiming the search for a final neurobiological cause (as in the other medical disciplines) (2). According to a realistic view, validity depends on how the theoretical constructs correspond to the external reality as it is, and the aim is to gradually approach a single and irreversible solution (the search for a specific neurobiological cause). On the other side, according to an instrumental view, validity corresponds to the adequacy of the constructs to the reality as we see it and how we deal with it (not necessarily as it is) (3). In this second case, the establishment of constructs depends on the objectives and context, needing to be recalibrated according to the time and needs (2).

Among the main objections to realistic validity is the fact that it is not clear that psychiatric disorders are similar to physical illnesses, probably presenting conceptual specificities. Therefore, the methodology for their investigation and of proving they are real may also have to be specific (4).

Accordingly, the validation of mental disorders looking for their underlying neurobiological cause appears to be too simplistic. Historically, the validity of psychiatric disorders has been built on criteria such as clinical presentation, clinical evolution, distinctive features from other disorders, response to treatments, beyond the pursuit of its biological cause (5). In modern times, new ways have been proposed to explore the complex and multidimensional relationship between mental symptoms and neurobiological activity. Among them, we can highlight new versions of the translational model, which seek new strategies to find biological correlates of the psychiatric symptoms through neuroimaging techniques (6, 7). Moreover, the complex role of hermeneutics in shaping a neurobiological signal as mental symptom has been considered in models trying to reconcile neurobiology and humanities (8, 9).

Shaffner finds an integrative position (between realism and instrumentalism), stating that until we find the final truth (we do not know if we are going to meet it 1 day), we must have an intermediate position (10). One day, the neurobiological bases of the psychiatric classifications based on the clinical presentation can be discovered, or alternatively, these classifications can continue to be clinically useful while a very complex etiopathogenic system (and difficult to systematize) develops. We must be aware that this latter hypothesis may occur because there may be no linear correlation between the way things relate and group at a macroscopic (clinical) level and at the microscopic level (neurosciences) (11).

If biology is the clearest model used by David to support the requirement that etiological theories must be validated, the other example comes from the cognitive field: “e.g., stressing activating events X irrational beliefs,” “no activation events X irrational beliefs generates non-B (no symptoms).” Probably due to the short space dedicated to this point, this example is far from clear and its degree of validation questionable.

Finally, there is no space to discuss it but the idea that “patient preferences are important […] but […] patients can and should be educated to support a knowledge-based society” introduces an outdated paternalistic approach against current models of values-based practice (12).

Concluding, we agree that it is important to validate therapeutic strategies independently of testing their efficacy, focusing on how and why the treatment works. However this validation, at least in the area of psychiatric treatments (including psychotherapy), is not always based on linear etiopathogenic chains, and this makes testing activities more difficult: “The question of validity in psychiatry is probably not linear and should not be seen as such. The most correct attitude may be to look at it as having multiple perspectives that can be used according to specific objectives”[(2), p. 5].

## Author contributions

DT conceived and designed research. DT and MA wrote the first draft of the manuscript. MA did critical revision for important intellectual content. The manuscript has been read and approved by all the authors. There are no other persons who satisfied the criteria for authorship but are not listed. We further confirm that the order of authors listed in the manuscript has been approved by all of us.

### Conflict of interest statement

The authors declare that the research was conducted in the absence of any commercial or financial relationships that could be construed as a potential conflict of interest.
